# Association between Financial Hardship and Symptom Burden in Patients Receiving Maintenance Dialysis: A Systematic Review

**DOI:** 10.3390/ijerph18189541

**Published:** 2021-09-10

**Authors:** Marques Shek Nam Ng, Dorothy Ngo Sheung Chan, Qinqin Cheng, Christine Miaskowski, Winnie Kwok Wei So

**Affiliations:** 1 The Nethersole School of Nursing, Faculty of Medicine, The Chinese University of Hong Kong, Hong Kong, China; marquesng@cuhk.edu.hk (M.S.N.N.); chengqinqin@link.cuhk.edu.hk (Q.C.); winnieso@cuhk.edu.hk (W.K.W.S.); 2 Department of Physiological Nursing, School of Nursing, University of California, San Francisco, CA 94143, USA; chris.miaskowski@ucsf.edu

**Keywords:** chronic kidney failure, dialysis, financial stress, signs and symptoms, systematic review

## Abstract

Background: Many patients on maintenance dialysis experience financial hardship. Existing studies are mainly cost analyses that quantify financial hardship in monetary terms, but an evaluation of its impact is also warranted. This review aims to explore the definition of financial hardship and its relationship with symptom burden among patients on dialysis. Methods: A literature search was conducted in November 2020, using six electronic databases. Studies published in English that examined the associations between financial hardship and symptom burden were selected. Two reviewers independently extracted data and appraised the studies by using the JBI Critical Appraisal Checklists. Results: Fifty cross-sectional and seven longitudinal studies were identified. Studies used income level, employment status, healthcare funding, and financial status to evaluate financial hardship. While relationships between decreased income, unemployment, and overall symptom burden were identified, evidence suggested that several symptoms, including depression, fatigue, pain, and sexual dysfunction, were more likely to be associated with changes in financial status. Conclusion: Our findings suggest that poor financial status may have a negative effect on physical and psychological well-being. However, a clear definition of financial hardship is warranted. Improving this assessment among patients on dialysis may prompt early interventions and minimize the negative impact of financial hardship.

## 1. Introduction

Chronic kidney disease is an evolving health problem worldwide. Because its global prevalence is increasing, major increases in costs related to treatment and productivity loss are projected [1]. Approximately 700 million cases were reported in 2017, contributing to 35.8 million disability-adjusted life years. Stage-five chronic kidney disease, or end-stage kidney disease (ESKD), has a significant impact on healthcare systems, as well as the affected individuals. Approximately 2% to 3% of healthcare expenditure is directed toward the management of ESKD in many developed countries, and the demand for dialysis is expected to double by 2030 [2]. Nevertheless, patients with ESKD often experience financial hardship due to treatment costs and income loss related to decreased productivity [3]. Despite the availability of reimbursement and financial support in some settings, patients, especially those receiving dialysis over a prolonged period, are prone to the negative impacts of financial hardship. A greater understanding of this impact may help healthcare professionals respond proactively to the financial needs of patients with ESKD [4].

While a clear definition is lacking, studies often attribute financial hardship to high healthcare expenditure, low income levels, and unemployment associated with ESKD. The cost of treatments is a major source of financial hardship, especially in countries without universal healthcare coverage. While dialysis treatments are not sufficiently reimbursed in more than 20% of countries [5], patients in some low-income countries (e.g., Bangladesh and Cambodia) need to cover most of the costs as out-of-pocket expenses [6,7]. Moreover, patients often experience reduced productivity because of the demanding schedule of dialysis treatment and their disabilities [8]. As a result, a lower employment rate and, consequently, lower income are observed globally [9]. Given that survival rates of patients with ESKD are improving (i.e., an average life expectancy of 10.4 years from the time of diagnosis) [10,11], patients may experience financial hardship for many years. In addition, the functional status of these patients decreases over time, while dependency increases [12]. Their financial hardship may be exacerbated because of the need for additional healthcare services for extended periods of time.

Financial hardship may compel patients to deplete their savings, liquidate their assets, or incur debt to pay for daily necessities and healthcare services [13]. If personal resources are not sufficient to cope with the deteriorating financial conditions, some patients may choose to file for bankruptcy or withdraw from treatment [6]. The negative impacts of financial hardship on physical and psychological health have been reported in patients with other chronic illnesses [14,15,16]. Of note, in one review [17], financial hardship was associated with depression and anxiety in patients with cancer. Poorer economic status may lead to adverse outcomes, such as impaired quality of life and increased mortality risk [18,19]. Given the chronicity of ESKD, patients receiving long-term dialysis can be more vulnerable to the negative impacts of financial hardship. Early assessment and intervention may improve the livelihood of patients, prevent adverse outcomes, and reduce the costs of care.

Previous studies of financial hardship in patients with ESKD on dialysis are mainly cost analyses that quantify financial hardship in monetary terms, from the perspective of the healthcare system [20,21]. This definition only reflects the material burden and not the perception or impact of this hardship. The lack of a comprehensive definition of financial hardship can hinder the provision of supportive care [8]. Patients are required to justify their needs for financial assistance by providing different forms of proof. Access to financial support may be delayed. While information on financial hardship is deemed helpful for planning supportive services and making clinical decisions, the definition of this hardship and its actual impact on patients remain unclear. Of note, preliminary evidence suggests that chronic illness and its treatments, symptom burden (i.e., subjective burden associated with the prevalence, frequency, and severity of symptoms), and financial hardship form a vicious cycle that affects the well-being of patients [22]. Given that symptom burden is common among patients with ESKD [23], additional research is warranted to increase our knowledge of the relationship between financial hardship and this burden [4].

This review explores how financial hardship was studied in previous studies and the relationship between financial hardship and symptom burden among patients receiving maintenance dialysis. The findings may inform the development of supportive care services that address these financial hardships, including ongoing assessments and interventions to alleviate the impact on patients.

## 2. Materials and Methods

To identify and synthesize existing evidence, a systematic review was conducted based on the Preferred Reporting Items for Systematic Reviews and Meta-Analyses (PRISMA) 2020 Statement [24].

### 2.1. Search Methods

A literature search was conducted by using six electronic databases—namely PubMed, Allied and Complementary Medicine Database, Embase, MEDLINE, PsycINFO, and Scopus—to retrieve studies published from database conception to November 2020. Search strategies were developed based on the concepts associated with dialysis, financial hardship, and symptom burden. Relevant Medical Subject Headings (MeSHs), such as dialysis, hemodialysis, peritoneal dialysis, healthcare costs, employment, income, poverty, symptom assessment, signs, and symptoms, were incorporated in the search (Supplementary Material S1).

### 2.2. Search Outcomes

Studies were included in the analysis if they (1) involved patients who were diagnosed with ESKD (defined as estimated glomerular filtration rate < 15 mL/min/1·73 m^2^) and received any modality of maintenance dialysis (i.e., hemodialysis or peritoneal dialysis); (2) examined the relationship between financial hardship (employment, income, health expenditure, etc.) and any individual symptoms and/or symptom burden; (3) reported an association between financial hardship and symptom burden in the results section; and (4) had the full text available in English. Studies that included pediatric patients or analyzed data from a mixed sample that included non-dialysis patients and/or caregivers were excluded. Abstracts, editorials, protocols, and reviews were also excluded.

### 2.3. Data Abstraction and Synthesis

The first reviewer (M.S.N.N.) extracted the sample characteristics and key findings from the included studies, using a designated form. The second reviewer (Q.C.) confirmed the extracted information. Given the heterogeneity in methodologies and outcomes, a meta-analysis was not feasible. Findings from the included studies were integrated and presented narratively according to the guidelines of the PRISMA 2020 Statement (Supplementary Material S2) [24].

### 2.4. Quality Appraisal

Quality appraisal was conducted by two reviewers (M.S.N.N. and Q.C.), using the Joanna Briggs Institute (JBI) Critical Appraisal Checklists for Analytical Cross-Sectional Studies [25] and Case Series [26] as appropriate. A third reviewer (D.N.S.C.) reviewed the results and resolved any disagreements. The two appraisal checklists contain 8 to 10 items to assess bias in the study design and process. The appraiser may determine whether the study achieved each item (yes/no) or whether sufficient information was reported (unclear). The checklists are not intended to suggest a cut-off, but to offer a comprehensive evaluation of the potential bias that may influence data synthesis and interpretation.

## 3. Results

**Figure 1:**
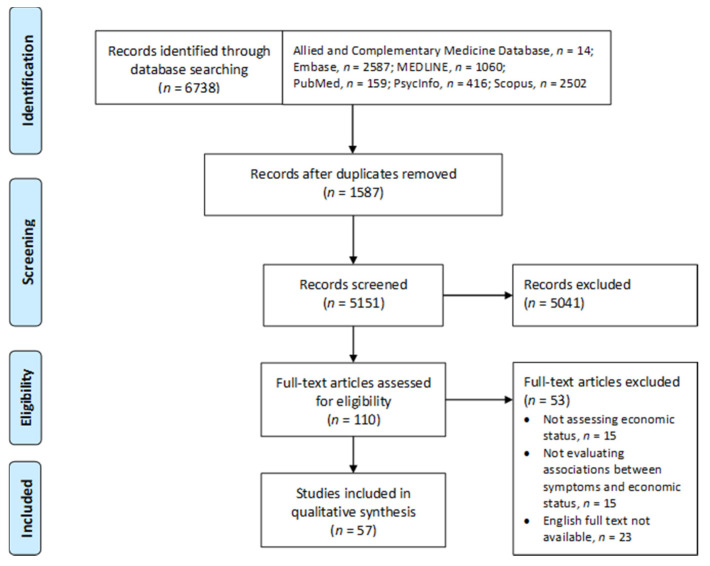
PRISMA flow diagram of study selection.

In total, 6738 records were identified from the electronic databases (Figure 1). After removing duplicates, the titles and abstracts of 5151 records were screened. Then, 110 records were selected and their full texts were retrieved to assess their eligibility. Of these, 53 studies did not meet the inclusion criteria and were excluded. Thus, 57 studies that fulfilled the pre-specified inclusion criteria were included in this review.

### 3.1. Study Characteristics

Among the included studies, seven were conducted in Mainland China [27,28,29,30,31,32,33], and five each were conducted in Brazil [34,35,36,37,38], Turkey [39,40,41,42,43], and the USA [44,45,46,47,48] (Supplementary Material S3). Nine studies were from the Middle East [49,50,51,52,53,54,55,56,57], and seven were from South Asian countries [58,59,60,61,62,63,64]. Two international studies that involved European and South American countries were identified [65,66]. The sample sizes ranged from 58 [44] to 28,561 [67]. While most of the studies only included patients on hemodialysis, in seven studies [29,31,32,48,68,69,70], 21.1% to 100% of the patients received peritoneal dialysis. Patients were predominantly male and had a mean age of 46.1 to 68.7 years.

While most studies used a cross-sectional design, seven were longitudinal studies with a follow-up period from 1 to 15 years [38,45,48,67,69,71,72]. Six studies evaluated the association between financial hardship and overall symptom burden. Other symptoms of interest included depression (*n* = 32), anxiety (*n* = 11), fatigue (*n* = 10), sexual dysfunction (*n* = 4), sleep disturbance (*n* = 4), pain (*n* = 3), constipation (*n* = 1), and itching (*n* = 1).

### 3.2. Quality Appraisal

The results of the quality appraisal are presented in Table 1 and Table 2. Twenty-six of the cross-sectional studies obtained a “yes” for seven out of eight items. Most of the studies (*n* ≥ 40) adopted standard criteria to define ESKD, identified potential confounding factors, and used appropriate statistical methods. Symptoms were assessed in a reliable and valid manner. However, the measurement of financial hardship was a major issue in these studies, because the reliability and/or validity of these measures are not well established. In addition, 17 studies did not provide details about the patients and/or settings. Twenty studies did not deal with potential confounding factors as part of their analyses.

Among the seven longitudinal studies, six obtained a “yes” for at least half of the 10 items. Patients’ demographic and clinical information, as well as their symptoms, were clearly reported in most of the studies (*n* ≥ 6). None of the studies achieved complete possible inclusion of patients. While one multi-center study in Japan included a large cohort of patients (*n* = 28,561), it did not specify whether all patients in the study sites were invited to participate in the study [67]. Information on consecutive sampling and details about study sites were not provided in most studies (*n* = 6).

### 3.3. Assessment of Financial Hardship

The studies included in the review mainly used four types of indicators to evaluate financial hardship, namely, income level, employment status, source of healthcare funding, and financial status (Table 3). Most of the studies used at least two of these indicators (*n* = 30). Twenty-nine studies evaluated the income level of patients. Most of them used predefined ranges to describe monthly or annual income. However, three studies did not specify the income period [56,60,72]. Some studies adopted national standards (e.g., average or quartiles of income and minimal wage) to classify income levels [35,52,53,67]. Two studies asked patients to determine whether they had experienced a budget deficit or surplus [43,64]. One study assessed the sources of income [36], and another inquired about subjective perceptions of income level [50].

Employment status was the most frequently used indicator of financial hardship among the included studies (*n* = 49). It is noteworthy that most of these indicators only reported employment status as a dichotomized variable, such as “employed” or “unemployed” (*n* = 27). Other studies provided various options to represent patients’ occupations. In two studies, employment experience was assessed by using instruments, namely the investigator-developed Job and Family Crisis Subscale [31] and the Kidney Disease Quality of Life (KDQOL) Work Status Subscale [44].

The source of healthcare funding was assessed in seven studies that were conducted in countries that relied on medical-insurance reimbursement (e.g., China). Four of these studies asked patients whether they were insured [29,32,47,56], and three studies required patients to indicate their major funding sources [33,58,60]. Finally, financial status was evaluated in eight studies. Six of these studies measured subjective perceptions of financial status by using terms such as “financial/economic status” [30,76,78], “financial problem” [57], “financial support” [59], or “difficulty in paying for basic needs” [48]. One study used the Modified Kuppuswamy Scale to differentiate social classes [58], and another study in Brazil adopted the national classification of socioeconomic status [38].

### 3.4. Associations with Financial Hardship

#### 3.4.1. Symptom Burden

Six studies evaluated the relationship between financial hardship and overall symptom burden. Symptom burden was assessed by using validated instruments, such as the Dialysis Symptom Index (DSI) and several quality-of-life measures. Among these studies, two did not identify any significant relationship [56,73]. One study found that income level indirectly affected symptom distress via the mediation of social support (*p* < 0.05) [27]. In one longitudinal study, a higher monthly income (i.e., >HK$20,000) was associated with a lower DSI score (*p* < 0.02) [69]. However, this association was not consistent over time.

Findings related to employment status were inconsistent. While one study in Pakistan reported that employed patients had a higher symptom burden, based on higher KDQOL symptom scores (*p* = 0.05) [60], two studies found associations between unemployment and a higher DSI score (*p* < 0.02) [53,69]. The reasons for these inconsistencies were not clear. However, the Pakistani study was the only study among the three that was conducted in a low- or middle-income country.

#### 3.4.2. Depression

Thirty-two studies examined the impact of financial hardship on depression. Among these studies, 12 found no significant relationship [31,35,36,37,44,48,57,70,72,77,80,81]. Studies found that a lower income was associated with an increased risk of depression (*n* = 4) [28,42,58,62] or a higher score on depression screening tests (e.g., Beck Depression Index [BDI] [49], Taiwanese Depression Questionnaire [71], and Patient Health Questionnaire [PHQ] [42]) (*n* = 3). A higher risk of depression among patients with a lower income was found over time in cohorts in a Japanese multi-center study (*p* < 0.05) [67].

Other studies examined the relationship between employment status and depression. The lack of a paying job was associated with a higher risk of depression (*n* = 6) [34,42,47,63,72,75] and a higher score on depression screening tests (e.g., BDI [34,74], PHQ [47], and Hospital Anxiety and Depression Scale [68,76]) (*n* = 5). In one study [55], while the incidence of depression was higher in employed patients than in unemployed patients (*p* = 0.04), employed patients had higher overall BDI scores (*p* = 0.03). This finding suggests that despite their lower risk, employed patients are vulnerable to the impact of depression.

In addition to income and employment, other factors that were related to a higher risk of depression included inadequate medical insurance coverage [58] and a lower financial status or level of financial support [59,78].

#### 3.4.3. Anxiety

Eleven studies assessed the relationship between financial hardship and anxiety. In most studies (*n* = 7), no significant relationship between financial hardship and anxiety was identified [31,36,37,57,68,70,72,81]. Other studies found that unemployment, lower income, and worse economic status were associated with an increase in the incidence of anxiety. In two studies, patients who were not employed showed higher State-Trait Anxiety Inventory trait scores (*p* = 0.02) [76] or Depression, Anxiety and Stress Scale 21 anxiety/stress scores (*p* < 0.001) [79]. One study in India reported that a monthly income < 5000 rupees was associated with an increased incidence of anxiety (*p* = 0.02) [61]. While a review also reported contrasting findings on the relationship between sociodemographic factors and anxiety [84], the reasons for these inconsistencies remain not clear.

#### 3.4.4. Fatigue

Among the 10 studies that evaluated fatigue severity, one found no significant relationship with financial hardship [45]. In one study [33], a lower monthly income (<RMB 900) was associated with higher Piper Fatigue Scale (PFS) mental, physical, and overall scores (all *p* < 0.001).

While employment status was another significant factor associated with fatigue, two studies did not report specific details [46,50]. In five other studies, unemployment was associated with a higher level of fatigue, as measured by the Fatigue Scale for Hemodialysis Patients [82], PFS [33,40], Fatigue Assessment Scale [83], and Visual Analogue Scale for Fatigue [41]. However, in one study [30], unemployed patients or patients with a lower economic status reported a lower level of fatigue (i.e., a higher Functional Assessment of Chronic Illness Therapy-Fatigue score; *p* ≤ 0.006). However, after adjusting for other covariates, these relationships were not significant.

#### 3.4.5. Sexual Dysfunction

Four studies examined sexual dysfunction and identified its relationship with financial hardship. Female patients who were housewives, retired, or unemployed reported a higher risk of sexual dysfunction [54,66] or lower scores for sexual arousal and orgasm, using the Female Sexual Function Index [65]. In two studies [54,60], patients with a lower income level reported worse sexual performance.

#### 3.4.6. Sleep Problems

One study identified a relationship between a lower income level (i.e., income less than outgoing) and poorer sleep quality, as measured by the Pittsburgh Sleep Quality Index (*p* < 0.001) [64]. However, in another study [62], these patients were less susceptible to sleep apnea (*p* = 0.027). Two studies did not draw any conclusions about the relationships between sleep problems and financial hardship [51,80]. These inconsistencies may be explained by the different aspects of sleep assessed.

#### 3.4.7. Pain

Three studies assessed associations with pain. A below-average income was associated with the presence of pain (*p* = 0.02) [52]. Patients with a lower socioeconomic status had higher pain subscale scores on Short Form 36 (*p* = 0.01) [38]. Findings regarding employment status were inconsistent. While in one study [60], patients who were not employed reported a lower level of pain (*p* = 0.49), unemployment was associated with a higher pain intensity score in another study (*p* = 0.001) [52].

#### 3.4.8. Itching

One study used the 5-D Itch Scale to evaluate the duration, degree, direction, distribution, and disability dimensions of itching [39]. Unemployed patients were reported to have a significantly higher score for the duration dimension (*p* = 0.01).

## 4. Discussion

This review is the first of its kind to examine the relationships between financial hardship and symptom burden among patients receiving maintenance dialysis. The association of financial hardship with treatment costs and reduced productivity among patients with ESKD has been documented [3], and so has the association of lower socioeconomic status with impaired quality of life and increased mortality [18,19]. However, little information is available about the impact of financial hardship on patients’ well-being. Our findings suggest that a poor financial status has a negative impact on patients’ physical and psychological symptoms. Therefore, more attention to financial hardship is warranted in renal care settings to improve the overall well-being of patients.

While relationships between decreased income, unemployment, and overall symptom burden were identified, considerable evidence suggests that several symptoms, including depression, fatigue, pain, and sexual dysfunction, were more likely to be associated with changes in financial status. These findings differ from those of previous studies in cancer patients, in which precise psychological symptoms (e.g., depression) were found to be affected [17]. The reasons for these discrepancies are not clear. Given the progressive nature of kidney disease, most patients may experience disease-related psychological distress, as well as physical deterioration [85]. In addition, the differences in study findings may be partly explained by the impaired physical health of patients who require dialysis. Patients who report a higher symptom burden may have higher levels of dependency or an increased need for healthcare services. Because of the increased costs of care and the reduced productivity, these patients are at a higher risk of financial hardship. Furthermore, the associations between financial hardship and symptom burden may be a consequence of health disparities. Patients experiencing financial hardship have fewer resources to meet their daily necessities and healthcare needs [86]. For example, higher mortality rates were found among patients with a lower socioeconomic status [19]. However, given the limited evidence, these hypotheses warrant additional research.

Psychosocial stress is an important factor that contributes to depression and sexual dysfunction among patients receiving dialysis [87,88]. As found in a study of patients with chronic illness [89], a deterioration in financial status may compel patients to withdraw from their usual social activities that they cannot afford. In addition, financial hardships may create challenges in fulfilling social roles, such as taking care of family members or pursuing personal goals [90]. Therefore, financial hardships may increase psychosocial stress and lead to depression and sexual dysfunction.

Fatigue and pain are two common physical symptoms reported by more than 60% of patients on dialysis [23]. In addition to their prevalence, their relationships with financial hardship were demonstrated by our findings. Because of the associated decrease in physical capacity, patients with these two symptoms have difficulties engaging in daily activities, including employment [91]. This eventually leads to income loss and a reduced ability to afford healthcare. Given the high prevalence of fatigue and pain, better symptom management is warranted to improve the quality of life and financial well-being of patients on dialysis.

Another important finding from this review is that the measurement of financial hardship is inconsistent. While studies generally used income, employment status, health, and the source of healthcare funding to evaluate an individual’s financial status, no standard definition exists for any of these indicators. For example, the ranges used to define income levels varied across studies. In addition, these indicators only reflect a single aspect of a patient’s financial status. Financial hardship is a much broader term that describes not only material shortages, but also the psychological responses and coping behaviors [14]. Addressing all of these aspects may alleviate some of the impact of ESKD and dialysis treatment on patients’ financial well-being and quality of life. In fact, very few studies included in this review assessed the “financial/economic status” or “financial problems” of patients [30,57,76]. However, these indicators lack conceptual clarity and only assess the objective perceptions of financial well-being. Recently, the concept of “financial toxicity” was introduced to reflect the subjective burden and objective stress associated with financial hardship, especially in patients with cancer [13]. The assessment of financial toxicity may provide a more comprehensive picture of financial well-being and prompt earlier interventions to prevent adverse outcomes [92].

In addition to the measurement of financial hardship, several gaps were identified that warrant investigation in future studies. First, while some studies identified the relationships between financial hardship and symptoms, the studies were focused on statistical associations and lacked a holistic perspective on these relationships. One study that used a mixed-methods approach to capture patients’ experiences about these associations found that those who were not employed experienced increased symptoms associated with physical exhaustion from housework [69]. Future studies should use mixed-methods approaches to explore these complex relationships and develop and test appropriate interventions. Second, in most of the included studies, financial condition was not the primary outcome but was used as a covariate in the analysis. Therefore, it is not clear how other factors that incur differences in treatment costs, such as dialysis modality [93], would influence the relationships with symptoms. Of note, the studies included in this review were conducted in regions with different health-financing arrangements. For example, while healthcare costs are covered by health insurance programs in China, Turkey, and the USA, a government-funded model is adopted in Brazil and many European countries [94]. These features of healthcare systems may cause differences in the experiences of financial hardship. However, given the limited evidence available, additional research is warranted to compare financial hardship across healthcare systems. Finally, evidence suggests that the care dependency of patients with ESKD increases over time [12]. While some studies used a longitudinal design to examine the temporal impact of financial hardship, the analyses were limited by the heterogeneous nature of the study group. To decrease the influence of different stages of the disease trajectory, a homogenous sample recruited at the initiation of dialysis treatment may be required to identify changes over time in the relationship between financial hardship and symptom burden.

### 4.1. Limitations and Recommendations

Some limitations of this review warrant consideration. First, only studies with the full text available in English were included. Because many studies were conducted in East Asian and South American countries, their results were published in the local language and were not reviewed. Databases with a comprehensive collection of these studies may be used in future studies. A collaborative effort is required to identify and review studies in various languages. Second, because of the considerable heterogeneity in settings, study designs, and measurements, the findings could only be integrated and presented narratively. Statistical pooling may be helpful to validate these relationships across studies. It should be noted that, while these findings are confined to specific methodologies and backgrounds (e.g., study population and healthcare financing arrangement), some studies provided incomplete descriptions about these confounders. These factors should be considered when interpreting the findings. In addition, this review aimed to describe the relationships between financial hardship and the symptoms that were reported in the included studies. While some reasons for these relationships are suggested based on the literature, the nature of these relationships warrants additional research.

### 4.2. Relevance to Clinical Practice

Findings from this review highlight the importance of strengthening financial assessments and support for patients receiving dialysis. Counseling and education are often provided to prepare patients for dialysis. An assessment of financial well-being should be performed before the commencement of dialysis treatment to help patients make informed choices about the dialysis modality and to make necessary long-term financial arrangements [95]. Ongoing assessments are warranted, because patients may experience changes in their financial status or care dependency at different stages of the disease trajectory [4]. Whenever a need is identified, healthcare professionals should initiate interventions to minimize the physical and psychological impacts of financial hardship. These interventions may include financial planning and arrangements for financial assistance. Furthermore, programs that offer training and support to assist patients to re-enter employment [96] should be used.

## 5. Conclusions

The financial hardship associated with treatment costs and reduced productivity among patients with ESKD on maintenance dialysis is significant. This hardship affects their daily life and has a negative impact on their physical and psychological health. Findings from this review suggest that relationships exist between different forms of financial hardship and overall symptom burden, depression, fatigue, pain, and sexual dysfunction. Improved assessments of financial hardship are warranted to capture its extent and impact on patients throughout the disease trajectory. Timely interventions may then help to prevent the harmful effects of financial hardship. Future research needs to focus on the measurement of financial hardship in renal care settings and the factors that influence patients’ financial status.

## Figures and Tables

**Table 1:** Methodological quality of cross-sectional studies.

Studies	Assessment Criteria ^1^								Number of Yeses
	1	2	3	4	5	6	7	8	
Anees et al., 2018 [60]	Y	U	Y	Y	Y	N	Y	Y	6
Dimova et al., 2019 [73]	Y	Y	Y	Y	Y	N	Y	Y	7
Fleishman et al., 2020 [53]	Y	Y	U	Y	Y	Y	Y	Y	7
Gao et al., 2016 [27]	Y	U	Y	Y	Y	Y	Y	Y	7
Karasneh et al., 2020 [56]	Y	U	Y	Y	Y	Y	Y	Y	7
Ahlawat, Tiwari, and D’Cruz, 2018 [58]	Y	U	Y	Y	Y	Y	Y	Y	7
AlShahrani et al., 2018 [49]	Y	U	Y	Y	Y	N	Y	U	5
Anees et al., 2008 [59]	U	U	U	Y	Y	Y	U	Y	4
Araujo et al., 2012 [34]	U	Y	U	Y	Y	Y	Y	Y	6
Čengić and Resić, 2010 [74]	U	Y	U	Y	Y	N	N	Y	4
de Alencar et al., 2020 [35]	Y	Y	Y	Y	Y	Y	U	Y	7
de Brito et al., 2019 [36]	Y	U	Y	Y	Y	N	Y	Y	6
Drayer et al., 2006 [44]	U	U	Y	Y	Y	Y	Y	Y	6
Ganu et al., 2018 [75]	Y	U	N	Y	Y	N	Y	Y	5
Gerogianni et al., 2018 [76]	Y	Y	U	Y	Y	Y	Y	Y	7
Hu et al., 2015 [28]	Y	Y	Y	Y	Y	Y	Y	Y	8
Ibrahim and Salamony, 2008 [55]	U	Y	U	Y	Y	Y	Y	Y	6
Jeon, Kim, and Kim, 2020 [77]	U	Y	Y	Y	Y	N	Y	Y	6
Kutner et al., 2010 [47]	Y	U	U	Y	Y	Y	Y	Y	6
Lai et al., 2005 [68]	Y	Y	U	Y	Y	N	Y	Y	6
Li et al., 2011 [29]	Y	Y	Y	Y	Y	N	Y	Y	7
Park et al., 2010 [78]	Y	Y	N	Y	Y	Y	Y	Y	7
Rai, Rustagi, and Kohli, 2011 [62]	Y	Y	Y	Y	Y	N	Y	Y	7
Ramirez et al., 2011 [37]	U	Y	Y	Y	Y	Y	Y	Y	7
Rebollo Rubio et al., 2017 [70]	Y	Y	U	Y	Y	N	Y	Y	6
Saeed et al., 2012 [63]	Y	U	Y	Y	Y	Y	Y	Y	7
Sezer et al., 2013 [42]	Y	Y	U	Y	Y	N	Y	Y	6
Sousa et al., 2019 [79]	Y	U	U	Y	Y	N	Y	Y	5
Tezel, Karabulutlu, and Şahin, 2011 [43]	Y	Y	Y	Y	Y	N	Y	Y	7
Trbojević-Stanković et al., 2014 [80]	Y	Y	U	Y	Y	N	Y	Y	6
Turkistani et al., 2014 [57]	Y	Y	U	Y	Y	Y	Y	Y	7
Ye et al., 2008 [31]	Y	Y	U	Y	Y	Y	Y	Y	7
Yoong et al., 2017 [81]	Y	Y	Y	Y	Y	Y	Y	Y	8
Mathews and Methew, 2017 [61]	U	U	Y	Y	Y	N	Y	Y	5
Bai et al., 2015 [82]	Y	Y	U	Y	Y	Y	Y	Y	7
Biniaz et al., 2013 [50]	Y	Y	U	Y	Y	N	Y	U	5
Jhamb et al., 2011 [46]	Y	U	U	Y	Y	Y	Y	Y	6
Karakan, Sezer, and Odemir, 2011 [40]	Y	U	Y	Y	Y	Y	Y	Y	7
Liu, 2006 [83]	Y	U	U	Y	Y	Y	Y	Y	6
Mollaoglu, 2009 [41]	Y	Y	U	Y	Y	Y	Y	Y	7
Wang et al., 2016 [30]	Y	Y	U	Y	Y	Y	Y	Y	7
Zuo et al., 2018 [33]	Y	U	Y	Y	Y	Y	Y	Y	7
Gatmiri et al., 2018 [54]	Y	Y	Y	Y	Y	N	Y	Y	7
Saglimbene et al., 2017 [65]	Y	Y	U	Y	Y	Y	Y	Y	7
Strippoli, 2012 [66]	Y	Y	U	Y	Y	Y	Y	Y	7
Einollahi et al., 2015 [51]	Y	Y	U	Y	Y	Y	Y	Y	7
Zubair and Butt, 2017 [64]	Y	Y	U	Y	Y	Y	Y	Y	7
Fleishman, Dreiher, and Shvartzman, 2018 [52]	Y	Y	U	Y	Y	Y	Y	Y	7
Zhang et al., 2013 [32]	Y	Y	U	Y	Y	N	Y	Y	6
Ersoy and Akyar, 2019 [39]	Y	Y	U	Y	Y	N	Y	Y	6
Number of studies with yes	35	31	15	42	42	25	40	41	

^1^ Quality appraisal was performed by using the JBI Critical Appraisal Checklists for Analytical Cross-Sectional Studies. The following eight criteria were included: (1) Were the criteria for inclusion in the sample clearly defined? (2) Were the study subjects and the setting described in detail? (3) Was the exposure measured in a valid and reliable way? (4) Were objective, standard criteria used for measurement of the condition? (5) Were confounding factors identified? (6) Were strategies to deal with confounding factors stated? (7) Were the outcomes measured in a valid and reliable way? (8) Was appropriate statistical analysis used? (Y = yes; N = no; U = unclear).

**Table 2:** Methodological quality of longitudinal studies.

Studies	Assessment Criteria ^1^										Number of Yeses
	1	2	3	4	5	6	7	8	9	10	
Ng et al., 2020 [69]	Y	Y	Y	U	N	Y	Y	Y	U	Y	7
Cheng, Ho, and Hung, 2018 [71]	N	N	N	U	U	Y	Y	Y	N	Y	4
Ng et al., 2015 [72]	Y	Y	Y	Y	U	Y	Y	Y	U	Y	8
Song et al., 2016 [48]	Y	N	Y	U	N	Y	Y	N	U	Y	5
Sugisawa et al., 2016 [67]	N	N	N	N	N	Y	Y	Y	Y	Y	5
Jhamb et al., 2009 [45]	Y	Y	Y	U	U	Y	U	Y	U	Y	6
Sesso, Rodrigues-Neto, and Ferraz, 2003 [38]	Y	Y	Y	U	U	Y	Y	Y	U	Y	7
Number of studies with yes	5	4	5	1	0	7	6	6	1	7	

^1^ Quality appraisal was performed by using the JBI Critical Appraisal Checklists for Case Series. The following 10 items were included: (1) Were there clear criteria for inclusion in the case series? (2) Was the condition measured in a standard, reliable way for all participants included in the case series? (3) Were valid methods used for identification of the condition for all participants included in the case series? (4) Did the case series have consecutive inclusion of participants? (5) Did the case series have complete inclusion of participants? (6) Was there clear reporting of the demographics of the participants in the study? (7) Was there clear reporting of clinical information of the participants? (8) Were the outcomes or follow-up results of cases clearly reported? (9) Was there clear reporting of the presenting site(s)/clinic(s) demographic information? (10) Was statistical analysis appropriate? (Y = yes; N = no; U = unclear).

**Table 3:** Assessment of financial hardship.

Studies	Income Level	Employment Status	Source of Healthcare Funding	Financial Status
Anees et al., 2018 [60]	Specific ranges (period not specified)	Multiple choices	Sources of funding: Multiple choice	
Dimova et al., 2019 [73]		Multiple choices		
Fleishman et al., 2020 [53]	Below/above average	Multiple choices		
Gao et al., 2016 [27]	Specific ranges			
Karasneh et al., 2020 [56]		Yes/no	Insurance: Yes/no	
Ng et al., 2020 [69]	Specific ranges	Multiple choices		
Ahlawat, Tiwari, and D’Cruz, 2018 [58]	Specific ranges (currency not specified)	Multiple choices	Sources of funding: Multiple choice	Modified Kuppusamy Scale
AlShahrani et al., 2018 [49]	Specific ranges	Yes/no		
Anees et al., 2008 [59]				Details not provided
Araujo et al., 2012 [34]		Yes/no		
Čengić and Resić, 2010 [74]		Yes/no		
Cheng, Ho, and Hung, 2018 [71]	Specific ranges	Yes/no		
de Alencar et al., 2020 [35]	Minimum monthly salary			
de Brito et al., 2019 [36]	Sources of income	Yes/no		
Drayer et al., 2006 [44]		Kidney Disease Quality of Life—Short Form		
Ganu et al., 2018 [75]	Details not provided	Multiple choices		
Gerogianni et al., 2018 [76]		Multiple choices		Perceived levels
Hu et al., 2015 [28]	Specific ranges			
Ibrahim and Salamony, 2008 [55]		Dichotomized responses		
Jeon, Kim, and Kim, 2020 [77]	Specific ranges	Multiple choices		
Kutner et al., 2010 [47]		Dichotomized responses	Employer group health insurance, disability income: Yes/no	
Lai et al., 2005 [68]		Multiple choices		
Li et al., 2011 [29]	Amount of annual income	Dichotomized responses	Reimbursement: Yes/no	
Ng et al., 2015 [72]	Specific ranges (period not specified)	Dichotomized responses		
Park et al., 2010 [78]				Perceived levels
Rai, Rustagi, and Kohli, 2011 [62]	Specific ranges	Yes/no		
Ramirez et al., 2011 [37]	Amount of monthly income			
Rebollo Rubio et al., 2017 [70]		Multiple choices		
Saeed et al., 2012 [63]	Specific ranges	Yes/no		
Sezer et al., 2013 [42]	Perceived levels	Yes/no		
Song et al., 2016 [48]	Specific ranges			Difficulty in paying for basic needs
Sousa et al., 2019 [79]		Dichotomized responses		
Sugisawa et al., 2016 [67]	Quartiles of annual income			
Tezel, Karabulutlu, and Şahin, 2011 [43]	Income-expenditure balance	Yes/no		
Trbojević-Stanković et al., 2014 [80]		Yes/no		
Turkistani et al., 2014 [57]		Multiple choices		Financial problems: Yes/no
Ye et al., 2008 [31]		Job and Family Crisis Subscale		
Yoong et al., 2017 [81]	Specific ranges	Multiple choices		
Mathews and Methew, 2017 [61]	Specific ranges	Multiple choices		
Bai et al., 2015 [82]		Yes/no		
Biniaz et al., 2013 [50]	Perceived levels	Details not reported		
Jhamb et al., 2009 [45]		Yes/no		
Jhamb et al., 2011 [46]		Yes/no		
Karakan, Sezer, and Odemir, 2011 [40]	Specific ranges (period not specified)	Multiple choices		
Liu, 2006 [83]		Yes/no		
Mollaoglu, 2009 [41]		Yes/no		
Sesso, Rodrigues-Neto, and Ferraz, 2003 [38]		Multiple choices		Brazilian classification of socioeconomic status
Wang et al., 2016 [30]		Yes/no		
Zuo et al., 2018 [33]			Medical expenses: Multiple choices	
Gatmiri et al., 2018 [54]	Specific ranges	Dichotomized responses		
Saglimbene et al., 2017 [65]		Multiple choices		
Strippoli, 2012 [66]		Multiple choices		
Einollahi et al., 2015 [51]		Multiple choices		
Zubair and Butt, 2017 [64]	Income-expenditure balance	Yes/no		
Fleishman, Dreiher, and Shvartzman, 2018 [52]	Below/above average	Yes/no		
Zhang et al., 2013 [32]		Yes/no	Insurance: Yes/no	
Ersoy and Akyar, 2019 [39]		Yes/no		

## Data Availability

The data presented in this study are available in this article and its supplementary material.

## Supplementary Materials

The following are available online at https://www.mdpi.com/article/10.3390/ijerph18189541/s1. Supplementary Material S1: Search strategy and results. Supplementary Material S2: Checklist of preferred reporting items. Supplementary Material S3: Characteristics and findings of the included studies.
