# Supplementary material for: Association between Financial Hardship and Symptom Burden in Patients Receiving Maintenance Dialysis: A Systematic Review
Source: Int J Environ Res Public Health. 2021 Sep 10;18(18):9541. doi: 10.3390/ijerph18189541 (PMC8464840; doi:10.3390/ijerph18189541)
Supplement: Supplementary file 1 [file ijerph-18-09541-s001.zip › IJERPH_S1 Search strategy_210908.pdf]

**Association between Financial Hardship and Symptom Burden in Patients Receiving Maintenance Dialysis: A Systematic Review**

Marques Shek Nam Ng, Dorothy Ngo Sheung Chan, Qinqin Cheng, Christine Miaskowski and Winnie Kwok Wei So

Supplementary Material S1: Search Strategy and Results

*Review Question*

What is the relationship between financial hardship and symptom burden among patients receiving maintenance dialysis?

*Date of Conducting Search*

25 November 2020

*Time Period for Search*

From conception to November 2020

*Concept Grid*

| Concepts | Dialysis | Financial hardship | Symptom burden |
|---|---|---|---|
| **MeSH** | Dialysis<br>Renal Dialysis<br>Peritoneal Dialysis<br>Peritoneal Dialysis, Continuous Ambulatory<br>Hemodialysis, Home | Economics<br>Cost of Illness<br>Health Care Costs<br>Health Expenditures<br>Financing, Personal<br>Employment<br>Income<br>Poverty | Symptom Assessment<br>Signs and Symptoms<br>Behavioral Symptoms<br>Affective symptoms |
| **Keywords** | dialysis OR peritoneal dial* OR h?emodial* OR HD OR PD OR CAPD OR APD OR IPD | expense* OR expenditure* OR cost* OR out?of?pocket OR co-payment* OR bankrupt* OR debt* OR credit* OR productivity OR employ* OR job* OR vocation* OR income* OR wage* OR salar* OR financial burden* OR economic burden* OR financial hardship* OR financial difficult* OR financial distress | symptom* OR symptom management OR symptom burden OR symptom occurrence OR symptom distress OR symptom severity OR symptom experience* OR symptom frequenc* |

*Search Strategy #1*

(Dialysis [MH] OR Renal Dialysis [MH] OR Peritoneal Dialysis [MH] OR Peritoneal Dialysis, Continuous Ambulatory [MH] OR Hemodialysis, Home [MH]) AND (Economics [MH] OR Cost of Illness [MH] OR Health Care Costs [MH] OR Health Expenditures [MH] OR Financing, Personal [MH] OR Employment [MH] OR Income [MH] OR Poverty [MH]) AND (Symptom Assessment [MH] OR Signs and Symptoms [MH] OR Behavioral Symptoms [MH] OR Affective symptoms [MH])

| Databases | No. of records |
|---|---|
| PubMed | 159 |

*Search Strategy #2*

1. (Dialysis OR Renal Dialysis OR Peritoneal Dialysis OR Peritoneal Dialysis, Continuous Ambulatory OR Hemodialysis, Home).ti,ab.
2. (dialysis or peritoneal dial* or h?emodial* or HD or PD or CAPD or APD or IPD).ti,ab.
3. (Economics OR Cost of Illness OR Health Care Costs OR Health Expenditures OR Financing, Personal OR Employment OR Income OR Poverty).ti,ab.
4. (expense* OR expenditure* OR cost* OR out?of?pocket OR bankrupt* OR debt* OR credit* OR productivity OR employ* OR job* OR vocation* OR income* OR wage* OR salar*).ti,ab.
5. (financial burden* OR economic burden* OR financial hardship* OR financial difficult* OR financial distress).ti,ab.
6. (Symptom Assessment OR (Signs and Symptoms) OR Behavioral Symptoms OR Affective symptoms).ti,ab.
7. (symptom* OR symptom management OR symptom burden OR symptom occurrence OR symptom distress OR symptom severity OR symptom experience* OR symptom frequenc*).ti,ab.

| Databases | No. of records |
|---|---|
| AMED | 14 |
| Embase | 2587 |
| MEDLINE | 1060 |
| PsycINFO | 416 |

*Search Strategy #3*

TITLE-ABS-KEY((Dialysis OR Renal Dialysis OR Peritoneal Dialysis OR Peritoneal Dialysis, Continuous Ambulatory OR Hemodialysis, Home) OR (dialysis or peritoneal dial* or h?emodial* or HD or PD or CAPD or APD or IPD)) AND ((Economics OR Cost of Illness OR Health Care Costs OR Health Expenditures OR Financing, Personal OR Employment OR Income OR Poverty) OR (expense* OR expenditure* OR cost* OR out?of?pocket OR bankrupt* OR debt* OR credit* OR productivity OR employ* OR job* OR vocation* OR income* OR wage* OR salar*) OR (financial burden* OR economic burden* OR financial hardship* OR financial difficult* OR financial distress)) AND ((Symptom Assessment OR (Signs and Symptoms) OR Behavioral Symptoms OR Affective symptoms) OR (symptom* OR symptom management OR symptom burden OR symptom occurrence OR symptom distress OR symptom severity OR symptom experience* OR symptom frequenc*))

| **Databases** | **No. of records** |
|---|---|
| Scopus | 2502 |
