# Supplementary material for: Association between Financial Hardship and Symptom Burden in Patients Receiving Maintenance Dialysis: A Systematic Review
Source: Int J Environ Res Public Health. 2021 Sep 10;18(18):9541. doi: 10.3390/ijerph18189541 (PMC8464840; doi:10.3390/ijerph18189541)
Supplement: Supplementary file 1 [file ijerph-18-09541-s001.zip › IJERPH_S3 Study characteristics_210908.pdf]

Association between Financial Hardship and Symptom Burden in Patients Receiving Maintenance Dialysis: A Systematic Review

Marques Shek Nam Ng, Dorothy Ngo Sheung Chan, Qinqin Cheng, Christine Miaskowski and Winnie Kwok Wei So

Supplementary Material S3: Characteristics and Findings of the Included Studies

Studies	Designs & objectives	Participants	Financial status outcomes	Symptom outcomes	Key findings
Symptom burden					
Anees et al., 2018 [60]<br><br>Pakistan	Cross-sectional<br><br>To emphasize the importance of socioeconomic factors affecting HRQOL	N=135<br>Sex: Not reported<br>Age: Not reported<br>Modality: HD (100%)<br>Time on dialysis: Not reported	Employment: Employed (24.6%), unemployed (57.7%), housewife (17.7%)<br>Income (duration not specified, Rs): ≤5,000 (3.8%), 5,000-25,000 (76.2%), >25,000 (20.0%)<br>Funding: Self (13.1%), family support (13.1%), government (65.4%), other (8.5%)	Symptom burden: Kidney Disease Quality of Life (KDQOL) - Symptoms of kidney disease subscale<br>· KDQOL symptoms of kidney disease score: 78.51±14.36<br>Pain: KDQOL - Pain subscale<br>· KDQOL pain score: 52.23±26.78<br>Sexual dysfunction: KDQOL-Sexual function subscale<br>· KDQOL sexual function score: 70.14±25.51	1. Employed patients reported a significantly higher symptom burden (p=.05) and a higher level of pain (p=.049) compared with other groups.<br>2. Patients with the highest income level reported a higher level of sexual function (p=.029) compared with other groups.
Dimova et al., 2019 [73]<br><br>Bulgaria	Cross-sectional<br><br>To examine the reliability and validity of the Bulgarian version of the Missoula-VITAS Quality of Life Index-15	N=263<br>Sex: Male (56.3%)<br>Age: 61-70 (28.1%), 51-70 (22.1%), 41-50 (18.6%)<br>Modality: HD (100%)<br>Time on dialysis (year): 2-5 (42.6%)	Employment: Unemployed (4.8%), public sector employee (5.8%), private sector employee (10.1%), self-employed (2.9%), retired (75.4%), other (1.0%)	Symptom burden: Missoula-VITAS Quality of Life Index (MVQOLI) - Symptoms subscale<br>· MVQOLI symptoms score: 3.52±12.11	No relevant significant finding.
Fleishman et al., 2020 [53]<br><br>Israel	Cross-sectional<br><br>To assess the relationship between symptom burden and patient factors as well as functional status and depression	N=336<br>Sex: Male (66.7%)<br>Age: 63.9±14.8<br>Modality: HD (100%)<br>Time on dialysis (month): >24 (52.1%)	Employment: Unemployed (80.4%)<br>Income: Below average (59.8%), average (12.8%), above average (27.4%)	Symptom burden: Dialysis Symptom Index (DSI)<br>· DSI score: 54.8±29.8	Being unemployed was associated with the median and 75th-percentile of the DSI score (p<.02). However, they were not significant factors of the symptom severity score.
Gao et al., 2016 [27]<br><br>China (Mainland)	Cross-sectional<br><br>To test the hypothesized model that explained the relationships between symptom distress, social support, and hope	N=207<br>Sex: Not reported<br>Age: 60-69 (34.3%), 50-59 (28.5%), 40-49 (22.7%)<br>Modality: HD (100%)<br>Time on dialysis (month): 24-48 (45.4%)	Income (monthly, RMB): <1,500 (16.4%), 1,500-5,000 (61.4%), >5,000 (22.2%)	Symptom burden: Dialysis Symptom Index (DSI)<br>· DSI score: 34.08±18.30	Patients with a higher income level had better social support (path coefficient: .44, p<.05), and it was associated with a lower level of symptom distress (path coefficient: -.99, p<.05).

Karasneh et al., 2020 [56]<br><br>Jordon	Cross-sectional<br><br>To assess the clinical characteristics and symptomatology	N=620<br>Sex: Male (59.8%)<br>Age: 50.9±16.1<br>Modality: HD (100%)<br>Time on dialysis: Not reported	Employment: Yes (14.5%)<br>Medical insurance: Yes (90.6%)	Symptom burden: CKD Symptom Burden Index (CKD-SBI)<br>· CKD-SBI score: 29.6±16.8	No relevant significant finding.
Ng et al., 2020 [69]<br><br>China (Hong Kong)	Longitudinal - 12 months (mixed methods)<br><br>To identify factors that influenced symptom burden	N=271<br>Sex: Male (58.3%)<br>Age: 60.0±11.4<br>Modality: PD (79.4%), HD (20.6%)<br>Time on dialysis (month): 53.4±22.8	Employment: Employed (18.5%), unemployed (24.0%), retired (54.6%), others (3.0%)<br>Income (monthly, HK$): <3,000 (38.7%), 3,001-10,000 (21.8%), 10,001-20,000 (21.0%), 20,001-40,000 (12.5%), >40,000 (5.9%)	Symptom burden: Dialysis Symptom Index (DSI)<br>· DSI score: 31.7±20.5 to 34.3±22.8	1. Being not employed was correlated with a higher level of symptom burden (r=-.125, p=.016). The correlation was not consistent over time.<br>2. A higher income (i.e., >HK$20,000) was associated with a lower level of symptom burden (p<.02). The association was not consistent over time.
Depression					
Ahlawat, Tiwari, & D'Cruz, 2018 [58]<br><br>India	Cross-sectional<br><br>To determine the prevalence of depression and the factors affecting it	N=612<br>Sex: Male (52.9%)<br>Age: 54.3 (SD/range not reported)<br>Modality: HD (100%)<br>Time on dialysis: Not reported	Employment: Employed (52.9%), unemployed (14.8%), retired (32.2%)<br>Income (monthly, currency not specified): ≤20,000 (63.7%), >20,000 (36.3%)<br>Treatment funding: Yes (27.0%), no (73.0%)<br>Social class: Modified Kuppusamy Scale - Upper (22.7%), middle (58.3%), lower (18.3%)	Depression: Patient Health Questionnaire (PHQ-9)<br>· PHQ-9 score: 7.1±3.5<br>· Prevalence of depression (PHQ-9 ≥ 5): 44.1%	1. More patients without treatment funding (45%), the employed (45%) or retired (44%), and those having income of ≤20,000 (48%) reported depressive symptoms compared with other groups (p≤.02).<br>2. In multivariate analysis, those without treatment funding (OR: 2.6) and having income of ≤20,000 (OR: 1.6) were predictors of depression (p-values not reported).
AlShahrani et al., 2018 [49]<br><br>Saudi Arabia	Cross-sectional<br><br>To assess the prevalence and determinants of clinical depression and impaired work productivity	N=233<br>Sex: Male (78.5)<br>Age: ≥60 (39.9%), 40-60 (34.3%), 20-40 (25.8%)<br>Modality: HD (100%)<br>Time on dialysis: Not reported	Employment: Working (26.6%), not working (73.4%)<br>Income (monthly, currency not specified): <5,000 (44.2%), 5,000-10,000 (43.8%), >10,000 (12.0%)	Depression: Beck Depression Index-II (BDI-II)<br>· Prevalence of depression: 42.0%	A higher income was associated with a lower BDI-II score (OR: 1.023, p-value not reported).
Anees et al., 2008 [59]<br><br>Pakistan	Cross-sectional<br><br>To check the frequency of depression and its risk factors	N=89<br>Sex: Male (58.4%)<br>Age: 49 (range not reported)<br>Modality: HD (100%)<br>Time on dialysis: Not reported	Financial support: Details not provided	Depression: Beck Depression Index (BDI), Diagnostic and Statistical Manual of Mental Disorders (DSM-IV)<br>· BDI-II score: 19.64 (SD/range not reported)<br>· Prevalence of depression: 27.0%,	Increased financial support was associated with a lower chance of depression (OR: 0.254, p=.004).

				25.8%, 30.3% (mild, moderate, and severe)	
Araujo et al., 2012 [34]<br><br>Brazil	Cross-sectional<br><br>To identify depressive symptoms and to evaluate associated risk factors	N=400<br>Sex: Male (59%)<br>Age: 51.6±5.5<br>Modality: HD (100%)<br>Time on dialysis (year): 5.9±5.5	Employment: Employed (15.3%)	Depression: Beck Depression Index-II (BDI-II)<br>· Prevalence of depression (BDI-II ≥ 16): 19.3%	The proportion of employed patients was higher in the group without depressive symptoms (18.4%) compared with the group with depressive symptoms (5.2%, p<.005). It was associated with a lower BDI-II score in the multivariate analysis (p=.001).
Čengić & Resić, 2010 [74]<br><br>Bosnia & Herzegovina	Cross-sectional<br><br>To investigate sociodemographic factors and prevalence of depression and the relationship between depression and HRQOL	N=200<br>Sex: Male (61.5%)<br>Age: 45-65 (50%)<br>Modality: HD (100%)<br>Time of dialysis (year): 1-5 (69%)	Employment: Unemployed (87%)	Depression: Beck Depression Index (BDI)<br>· Prevalence of depression (BDI > 11): 51%	Unemployed patients reported a higher BDI score than those who were employed (p<.002).
Cheng, Ho, & Hung, 2018 [71]<br><br>China (Taiwan)	Longitudinal - 3 years<br><br>1. To determine how depressive symptoms affected 3-year mortality and HRQOL<br>2. To investigate the association between depressive symptoms and demographic and biochemical parameters	N=151<br>Sex: Female (50.1%)<br>Age: 46.6±13.9<br>Modality: HD (100%)<br>Time on dialysis (year): 4.5±3.9	Employment: Unemployed (87.4%), employed (16.3%)<br>Income (monthly, US$): <300 (57.6%), 300-1,000 (24.5%), 1,000-2,000 (11.3%), >2,000 (6.6%)	Depression: Taiwanese Depression Questionnaire (TDQ)<br>· Prevalence of depression (TDQ ≥ 19): 43.0%	Patients having income higher than US$1,000 reported a lower TDQ Somatic score than those who had less (p=.019).
de Alencar et al., 2020 [35]<br><br>Brazil	Cross-sectional<br><br>To identify the prevalence of depression, its associated factors, and HRQOL in an older population on HD	N=173<br>Sex: Male (58.4%)<br>Age: 68.7±6.9<br>Modality: HD (100%)<br>Time on dialysis (month): *34.5 (15-72)	Income (minimum monthly salary): 1 (14.4%), 1-5 (81.2%), >5 (4.4%)	Depression: Geriatric Depression Scale, Mini-International Neuropsychiatric Interview (MINI)<br>· Prevalence of depression: 43.3%	No relevant significant finding.

de Brito et al., 2019 [36]<br><br>Brazil	Cross-sectional<br><br>1. To investigate the prevalence of depression and anxiety<br>2. To investigate the factors associated with the presence and severity of depression and anxiety	N=205 (dialysis: n=130) [Overall]<br>Sex: Male (52.7%)<br>Age: 54.5±12.7<br>Modality: Not reported<br>Time on dialysis (month): 120.1±8.1	Employment: Yes (13.8%)<br>Income: Work (10.8%), benefits (78.5%)	Anxiety: Beck Anxiety Index (BAI)<br>· Prevalence of anxiety (BAI > 10): 31.5%<br>Depression: Beck Depression Index (BDI)<br>· Prevalence of anxiety (BDI > 11): 59.2%	No relevant significant finding.
Drayer et al., 2006 [44]<br><br>USA	Cross-sectional<br><br>To determine the rates, correlates, and outcomes of depression	N=58<br>Sex: Male (89.7%)<br>Age: 57 (18-91)<br>Modality: HD (100%)<br>Time on dialysis: Not reported	Employment: Kidney Disease Quality of Life-Short Form - Work Status subscale (2 questions about work)	Depression: Patient Health Questionnaire (PHQ-9)<br>· PHQ-9 score: 8.7±4.9<br>· Prevalence of depression (PHQ-9 ≥ 5): 29.3%	No relevant significant finding.
Ganu et al., 2018 [75]<br><br>Ghana	Cross-sectional<br><br>1. To examine the prevalence of depression and HRQOL status<br>2. To explore the impact of demographic characteristics on depression and HRQOL	N=106<br>Sex: Male (59.4%)<br>Age: 40-49 (27.4%), 50-59 (22.6%), 60-69 (19.8%)<br>Modality: HD (100%)<br>Time on dialysis: Not reported	Employment: Actively working (36.8%), not actively working (45.3%), pensioner (11.3%), student (6.6%)<br>Income: Details not reported	Depression: Patient Health Questionnaire (PHQ-9)<br>· Prevalence of depression: 26.42%, 12.26%, 56.6% (moderate, moderately severe, and severe)	Patients actively working reported less depressive symptoms than those who were not actively working (details not reported).
Gerogianni et al., 2018 [76]<br><br>Greece	Cross-sectional<br><br>To evaluate the prevalence of depression and anxiety and its association with sociodemographic factors	N=414<br>Sex: Male (63.3%)<br>Age: 63.5 (54.0-72.4)<br>Modality: HD (100%)<br>Time on dialysis (month): *36 (6-72)	Employment: Civil servant (3.1%), private employee (2.7%), freelancer (3.9%), household (10.1%), farmer (1.2%), student (0.7%), unemployed (2.4%), pensioner (75.8%)<br>Financial status: Bad (16.7%), moderate (56.0%), good (24.9%), very good (2.2%), perfect (0.2%)	Anxiety: Hospital Anxiety and Depression Scale (HADS) - Anxiety score, State-Trait Anxiety Inventory (STAI)<br>· HADS anxiety score: *6 (3-9)<br>· STAI score: *34.5 (27-46), *39 (30-47) (state and trait)<br>Depression: HADS - Depression score, Beck Depression Index (BDI)<br>· HADS depression score: *5 (2-8)<br>· BDI score: *13 (8-9)	1. Employed patients reported a lower STAI trait score than the pensioners or housekeepers (p=.02). Those with good/very good financial status reported a lower level of anxiety in terms of HADS anxiety, and STAI state and trait scores (p=.001).<br>2. Employed patients reported a lower BDI score (p=.001). Those with good/very good financial status reported a lower level of depression in terms of HADS depression and BDI scores (p=.001).
Hu et al., 2015 [28]<br><br>China (Mainland)	Cross-sectional<br><br>To explore the prevalence and treatment of major depressive disorder	N=260<br>Sex: Male (61.5%)<br>Age: 55-87 (80.8%), 40-54 (11.5%), 18-39 (7.7%)<br>Modality: HD (100%)<br>Time on dialysis (month): ≥13 (53.8%)	Income (monthly, RMB): <2,000 (65.4%), 2,000-4,000 (26.9%), >4,000 (7.7%)	Depression: Patient Health Questionnaire (PHQ-9)<br>· Prevalence of depression: 30.8%, 50.0%, 19.2% (moderate, severe, and very severe)	A higher monthly income was associated with a lower risk for major depressive disorder (OR: 0.164, p<.001).

Ibrahim & Salamony, 2008 [55]<br><br>Egypt	Cross-sectional<br><br>To assess the prevalence of depressive symptoms and HRQOL in order to identify those at high risk for poor outcome	N=60<br>Sex: Male (51.7%)<br>Age: 46.13±16.55<br>Modality: HD (100%)<br>Time on dialysis (months): 67.03±56.09	Employment: Employed full/part-time (20%), unemployed/retired (80%)	Depression: Beck Depression Index (BDI)<br>· Prevalence of depression (BDI ≥ 15): 33.3%	1. Among the employed patients, the proportion in the group without depressive symptom (20%) is higher than that in the group with depression symptom (0%, p=.04). However, this association was not significant in the multivariate analysis.<br>2. Employed patients reported a higher BDI score than those not employed (p=.03).
Jeon, Kim, & Kim, 2020 [77]<br><br>South Korea	Cross-sectional<br><br>To identify weather fatigue, social adaptation, and subjective health affect depressive symptoms	N=71<br>Sex: Male (87.3%)<br>Age: 49.30±10.58<br>Modality: HD (100%)<br>Time on dialysis (year): ≥3 (63.3%)	Employment: Business (26.8%), service job (18.3%), labor/farmer (9.9%), office worker/professional (32.4%), other (12.7%)<br>Income (monthly, US$): <2,000 (36.6%), 2,000-2,999 (31.0%), 3,000-3,999 (14.1%), ≥4,000 (18.3%)	Depression: Center for Epidemiological Studies-Depression Scale (CES-D)<br>· CES-D score: 17.61±10.64<br>· Prevalence of depression (CES-D ≥ 21): 32.4%	No relevant significant finding.
Kutner et al., 2010 [47]<br><br>USA	Cross-sectional<br><br>To test the hypothesis that usual activity level would be independently associated with employment maintenance after dialysis start	N=585<br>Sex: Male (55.0%)<br>Age: 59.6±14.2<br>Modality: HD (100%)<br>Time on dialysis: Not reported	Employment: Working now full or part time (32.6%), not working (67.4%)<br>Employer Group Health (EGH) insurance: 27.7%<br>Disability income: 38.9%	Depression: Patient Health Questionnaire Depression Module (PHQ-2)<br>· PHQ-2 score: 1.0±1.5, 1.8±1.9 (working and not working)<br>· Prevalence of depression (PHQ-2 ≥ 3): 12.1%, 32.8% (working and not working)	1. Employed patients reported a lower PHQ-2 score than those who were not working (p<.0001). Less employed patients have possible depression (12.1%) compared with those who were not working (32.8%, p<.0001).<br>2. Having EGH insurance (OR: 3.25, p<.0001) and receiving disability income (OR: 0.26, p<.0001) were associated with continued employed.
Lai et al., 2005 [68]<br><br>China (Hong Kong)	Cross-sectional<br><br>To explore the clinical factors associated with psychological problems and their potential implications	N=167<br>Sex: Female (52.1%)<br>Age: 60.7±13.4<br>Modality: PD (100%)<br>Time on dialysis (month): 42.9±40.1	Employment: Full-time job (6.6%), housewife (19.8%), retired (34.7%), unemployed (9.6%), not fit to work (29.3%)	Anxiety: Hospital Anxiety and Depression Scale (HADS) - Anxiety score<br>· Prevalence of anxiety (HADS anxiety ≥ 8): 19.8%<br>Depression: HADS - Depression score<br>· Prevalence of anxiety (HADS depression ≥ 8): 45.6%	Patients working full-time reported a lower HADS depression score (p=.011). No significant difference was found in anxiety score.

Li et al., 2011 [29]<br><br>China (Mainland)	Cross-sectional<br><br>To test the hypothesized association between depression and malnutrition-inflammation complex syndrome	N=142<br>Sex: Male (57.7%)<br>Age: 53 (18-87)<br>Modality: PD (100%)<br>Time on dialysis (month): 24.3 (range: 3-117.8)	Employment: Employed (59.1%), other (40.8%)<br>Reimbursement: Yes (79.6%), no (20.4%)<br>Income (annual, US$): 7,235±835 (non-depressed), 6,446±743 (depressed)	Depression: Hamilton Depression Rating Scale (HAMD)<br>· HAMD score: 7.12±5.28<br>· Prevalence of depression (HAMD ≥ 10): 26.1%	More employed patients did not report depressive symptoms (63.8%) compared with those who were not (36.2%, p=.047). More reimbursed patients did not report depressive symptoms (83.8%) compared with those who were not (16.2%, p=.040).
Ng et al., 2015 [72]<br><br>Singapore	Longitudinal - 12 months<br><br>1. To examine course of anxiety and depression over 12 months<br>2. To explore the frequency of a priori fixed trajectories over time<br>3. To examine the associations of these trajectories and different variables	N=159<br>Sex: Male (59.7%)<br>Age: 53.3±4.6<br>Modality: HD (100%)<br>Time on dialysis (month): 50.6±53.79	Employment: Part-time or full-time (46.7%)<br>Income (duration not specified, S$): ≤2,000 (44.4%), 2,001-4,000 (27.0%), 4,001-6,000 (4.4%), >6,000 (3.1%)	Anxiety: Hospital Anxiety and Depression Scale (HADS) - Anxiety score<br>· HADS anxiety score: 7.00±4.39, 7.00±4.70 (baseline and follow-up)<br>Depression: HADS - Depression score<br>· HADS depression score: 8.09±4.17, 8.15±7.11 (baseline and follow-up)	No relevant significant finding.
Park et al., 2010 [78]<br><br>South Korea	Cross-sectional<br><br>To determine the prevalence of depression and measure HRQOL	N=160<br>Sex: Male (61.9%)<br>Age: 56.9±13.8<br>Modality: HD (100%)<br>Time on dialysis (year): 5.8±5.3	Economic status: Low (25.6%), high (70.6%)	Depression: Korean Beck Depression Inventory (K-BDI)<br>· K-BDI score: 14.21±9.78<br>· Prevalence of depression (K-BDI ≥ 10): 31.3%	More patients with low financial status reported depression (48.8%) compared with those with high financial status (25.7%, p=.006). The association between depression and low financial status was significant in multivariate analysis (OR: 4.747, p=.002).
Rai, Rustagi, & Kohli, 2011 [62]<br><br>India	Cross-sectional<br><br>To find out the prevalence of depression and sleep problems and to correlate it with demographic profile	N=69<br>Sex: Male (68.1%)<br>Age: 52.82±8.61<br>Modality: HD (100%)<br>Time on dialysis (year): <1 (62.3%)	Employment: Yes (26.1%), no (73.9%)<br>Income (monthly, Rs): <5,000 (33.3%), 5,000-10,000 (39.1%), >10,000 (27.5%)	Depression: Beck Depression Inventory (BDI)<br>· Prevalence of depression (BDI > 15): 47.8%<br>Sleep apnea: Berlin Sleep Apnea Questionnaire (BSA)<br>· High risk for sleep apnea: 24.6%<br>Insomnia: Self-developed questionnaire<br>· Prevalence of insomnia: 60.9%	1. More patients with income <Rs5,000 reported depression (69.6%, p=.03) compared with other groups. More patients with income >Rs10,000 reported sleep apnea (47.4%, p=.027).<br>2. More patients who were not employed reported depression (54.9%) compared with those employed (27.8%, p=.009).

Ramirez et al., 2011 [37]<br><br>Brazil	Cross-sectional<br><br>To investigate the associations of positive religious coping and religious struggle with psychological distress and HRQOL	N=170<br>Sex: Male (64.1%)<br>Age: 48.4±14.2<br>Modality: HD (100%)<br>Time on dialysis (month): 65.7±64.8	Income (monthly, US$): 588±188	Anxiety: Hospital Anxiety and Depression Scale (HADS) - Anxiety score<br>· HADS anxiety score: 5.04±4.24<br>· Prevalence of anxiety (HADS anxiety ≥ 8): 25.9%<br>Depression: HADS - Depression score<br>· HADS depression score: 4.29±3.75<br>· Prevalence of depression (HADS depression ≥ 8): 15.3%	No relevant significant finding.
Rebollo Rubio et al., 2017 [70]<br><br>Spain	Cross-sectional<br><br>To analyze the HRQOL and the influence of anxiety and depression on HRQOL	N=152<br>Sex: Male (71.7%)<br>Age: 62.5±14.1<br>Modality: HD (78.9%), PD (21.1%)<br>Time on dialysis: Not reported	Employment: Employed (8.3%), disability (14.5%), pensioner (64.1%), unemployed (5.4%)	Anxiety: Hospital Anxiety and Depression Scale (HADS) - Anxiety score<br>· Prevalence of anxiety (HADS anxiety ≥ 8): 46.0%<br>Depression: HADS - Depression score<br>· Prevalence of depression (HADS depression ≥ 8): 40.1%	No relevant significant finding.
Saeed et al., 2012 [63]<br><br>Pakistan	Cross-sectional<br><br>To determine the frequency of depression in patients undergoing HD and their caregivers in order to delineate potential associated risk factors for both groups	N=360 (patient: n=180) [Patients]<br>Age: 48 (14-83)<br>Sex: Male (55.0%)<br>Modality: HD (100%)<br>Time on dialysis: Not reported	Employment: Employed (12.8%)<br>Income (monthly, Rs): <5,000 (63.9%), 5,000-10,000 (12.8%), 10,000-14,999 (7.8%), >15,000 (15.6%)	Depression: Beck Depression Index-II (BDI-II)<br>· BDI-II score: 25.4±11.4<br>· Prevalence of depression (BDI-II ≥ 20): 75%	Being employed (OR: 0.208, p=.01) was associated with lower chance of depression in multivariate analysis.
Sezer et al., 2013 [42]<br><br>Turkey	Cross-sectional<br><br>To analyze the factors the factors influencing depression scores of HD patients and their caregivers	N=141<br>Age: 52.91±14.56 (non-depressed), 55.02±13.83 (depressed)<br>Sex: Male (61.7%)<br>Modality: HD (100%)<br>Time on dialysis (year): *8.5 (11.25), *5 (12) (non-depressed and depressed)	Employment: Unemployed (85.8%)<br>Income (monthly): Low (6.4%), moderate (81.6%), high (12.1%)	Depression: Beck Depression Index-II (BDI-II)<br>· BDI-II score: *8, *24 (non-depressed and depressed, range not reported)<br>· Prevalence of depression (BDI-II ≥ 19): 36.2%	1. More unemployed patients reported depressive symptoms (39%) compared with those employed (16%, p=.049).<br>2. More patients with low income reported depressive symptoms (67.0%) compared with those with moderate and high income (36.5% and 17.6%, p=.046). Higher monthly income was correlated with a lower BDI score (r=-0.278, p<.005).

Song et al., 2016 [48]<br><br>USA	Longitudinal - 12 months<br><br>1. To describe depressive symptoms experienced by chronic dialysis patients on monthly assessments<br>2. To describe factors identified by these patients as contributing to their symptoms<br>3. To describe how they responded to the symptoms	N=210<br>Age: 59.1±12.4<br>Sex: Male (51.9%)<br>Modality: HD (94.8%)<br>Time on dialysis (year): 4.2±5.3	Income (annual, US$): <20,000 (51.9%), 20,000-50,000 (32.9%), >50,000 (11.0%)<br>Difficulty in paying for basic needs: Not at all/somewhat difficult (73.3%), very/extremely difficult (26.7%)	Depression: Center for Epidemiological Studies Depression Scale-Short Form (CESD-SF)<br>· Prevalence of depression (CESD-DF ≥ 10): 47.6%	No relevant significant finding. However, financial difficulties were reported by 31.0% of patients that contributed to the symptom burden.
Sousa et al., 2019 [79]<br><br>Portugal	Cross-sectional<br><br>To investigate the relationship between subjective well-being, sense of humor, and anxiety, depression, and stress with subjective happiness	N=183<br>Age: 59.17±14.64<br>Sex: Male (not reported)<br>Modality: HD (100%)<br>Time on dialysis (month): 70.9±54.2	Employment: Regular job (24%), retired (76%)	Anxiety: Depression, Anxiety and Stress Scale 21 (DASS-21) - Anxiety/stress subscale<br>· DASS-21 anxiety/stress score: 6.9±6.5<br>Depression: DASS-21 - Depression subscale<br>· DASS-21 score: 4.3±4.4	Being employed was correlated with a decreased DASS-21 score (σ=-0.222, p<.001).
Sugisawa et al., 2016 [67]<br><br>Japan	Longitudinal - 15 years<br><br>To examine whether socioeconomic status-related gaps in physical and mental health change with age, period, and cohort	N=28,561<br>[2011 survey]<br>Age: 50-59 (22.3%), 60-69 (41.6%), ≥70 (25.3%)<br>Sex: Male (55.2%)<br>Modality: HD (100%)<br>Time on dialysis (month): >15 (33.1%)	Income (annual): First quartile (24.5%), second/third quartile (44.5%), fourth quartile (21.1%)	Depression: Center for Epidemiological Studies-Depression Scale (CES-D)<br>(Outcome details not provided)	1. The mean differences in depressive symptoms were positive associated with having first quartile of income (coefficient: 1.018, p=.012).<br>2. The mean differences in depressive symptoms were negatively associated with the interaction between age of 50-59 years and first quartile of income (coefficient: -0.477, p=.033).<br>3. The mean differences in depressive symptoms were positive associated with the interactions between cohorts (2006, 2011) and different levels of income (coefficients: 0.813-1.180, p<.05).
Tezel, Karabulutlu, & Şahin, 2011 [43]<br><br>Turkey	Cross-sectional<br><br>To determine the perceived social support from family and depression level	N=147<br>Age: >50 (44.9%), 41-50 (19.7%), 31-40 (18.4%)<br>Sex: Female (57.1%)<br>Modality: HD (100%)<br>Time on dialysis (year): ≥3 (36.7%)	Employment: Employed (13.6%), unemployed (86.4%)<br>Income: income>expenditure (8.2%), income=expenditure (6.8%), income<expenditure (85.0%)	Depression: Beck Depression Index (BDI)<br>· BDI score: 23.2±10.5	Unemployed patients reported a higher BDI score than those who were employed (p=.022).

Trbojević-Stanković et al., 2014 [80]<br><br>Serbia	Cross-sectional<br><br>To determine the prevalence of depression and poor sleep quality and to example the association between these disorders and demographic, clinical, and treatment-related characteristics	N=222<br>Age: 57.3±11.9<br>Sex: Male (59.5%)<br>Modality: HD (100%)<br>Time on dialysis (month): 61.4±60.3	Employment: Employed (1.8%), unemployed (98.2%)	Depression: Beck Depression Index (BDI)<br>· BDI score: 16.1±11.3<br>· Prevalence of depression (BDI ≥ 14): 49.1%<br>Sleep: Pittsburgh Sleep Quality Index (PSQI)<br>· PSQI score: 7.8±4.5<br>· Prevalence of depression (PQSI > 5): 64.2%	No relevant significant finding.
Turkistani et al., 2014 [57]<br><br>Saudi Arabia	Cross-sectional<br><br>To screen for prevalence of depression and anxiety and to investigate the effect of sociodemographic characteristics on the prevalence of depression and anxiety	N=286<br>Sex: Male (58.2%)<br>Age: >40 (68.8%), 20-40 (27.7%), <20 (3.5%)<br>Modality: HD (100%)<br>Time on dialysis: Not reported	Employment: Yes (16.3%), no (56.5%), retired (27.7%)<br>Financial problem: Yes (32.2%), no (56.5%)	Anxiety: Hospital Anxiety and Depression Scale (HADS) - Anxiety score<br>· Prevalence of anxiety (HADS anxiety ≥ 8): 39.6%<br>Depression: HADS - Depression score<br>· Prevalence of depression (HADS depression ≥ 8): 44.8%	No relevant significant finding.
Ye et al., 2008 [31]<br><br>China (Mainland)	Cross-sectional<br><br>To examine the major effects of psychological stress and social support on anxiety and depressive symptoms	N=81<br>Sex: Male (60.49%)<br>Age: 48.9±16.3<br>Modality: PD (100%)<br>Time on dialysis: Not reported	Employment: Self-developed questionnaire - Job and Family Crisis subscale (4 of 6 questions about work)	Anxiety: Hospital Anxiety and Depression Scale (HADS) - Anxiety score<br>Depression: HADS - Depression score<br>(Outcome details not provided)	No relevant significant finding.
Yoong et al., 2017 [81]<br><br>Singapore	Cross-sectional<br><br>To document and compare prevalence rates of anxiety and depression between ESRD patients with and without coexisting DM	N=526<br>Sex: Male (59%)<br>Age: 56.1±10.8<br>Modality: HD (100%)<br>Time on dialysis (month): 52.9±53.2	Employment: Employed/student (28%), unemployed (24%), retired (19%), looking after home and family (15%), others (14%)<br>Income (monthly, S$): 0-2,000 (49%), 2,001-4,000 (24%), 4,001-6,000 (5%), ≥6,001 (3%)	Anxiety: Hospital Anxiety and Depression Scale (HADS) - Anxiety score<br>· HADS anxiety score: 7.28±4.15<br>· Prevalence of anxiety (HADS anxiety ≥ 8): 45.4%<br>Depression: HADS - Depression score<br>· HADS depression score: 7.81±4.23<br>· Prevalence of depression (HADS depression ≥ 8): 49.9%	No relevant significant finding.
Anxiety					
de Brito et al., 2019 [36]	Please refer to 'depression'				
Gerogianni et al., 2018 [76]	Please refer to 'depression'				

Lai et al., 2005 [48]	Please refer to 'depression'				
Mathews & Methew, 2017 [61]<br><br>India	Cross-sectional<br><br>1. To determine the level of anxiety<br>2. To determine the association between anxiety and selected demographic and socioeconomic characteristics<br>3. To determine the association between anxiety and selcted disease/treatment-related characteristics	N=112<br>Sex: Male (73.2%)<br>Age: >40 (95.5%)<br>Modality: HD (100%)<br>Time on dialysis: Not reported	Employment: Manual worker (19.6%), business (7.1%), employed (36.6%), unemployed (36.6%)<br>Income (monthly, Rs): <1,000 (7.8%), 1,000-5,000 (55.9%), >5,000 (46.0%)	Anxiety: Beck Anxiety Index (BAI)<br>· Prevalence of anxiety: 21.4%, 47.3%, 31.3% (mild, moderate, and severe)	Patients having income less than Rs5,000 reported a higher level of anxiety (p=.02).
Ng et al., 2015 [72]	Please refer to 'depression'				
Ramirez et al., 2011 [37]	Please refer to 'depression'				
Rebollo Rubio et al., 2017 [70]	Please refer to 'depression'				
Sousa et al., 2019 [79]	Please refer to 'depression'				
Turkistani et al., 2014 [57]	Please refer to 'depression'				
Ye et al., 2008 [31]	Please refer to 'depression'				
Yoong et al., 2017 [81]	Please refer to 'depression'				
Fatigue					
Bai et al., 2015 [82]<br><br>China (Taiwan)	Cross-sectional<br><br>To investigate the fatigue level and to analyze the predictors that affect fatigue	N=193<br>Sex: Female (51.8%)<br>Age: 61.17±12.71<br>Modality: HD (100%)<br>Time on dialysis (month): 72.92±59.49	Employment: Yes (30.6%), no (69.4%)	Fatigue: Fatigue Scale for Hemodialysis Patients<br>· Fatigue Scale score: 49.40±17.92	Being employed was associated with a lower fatigue score compared with those not employed in multivariate analysis (p<.001).
Biniaz et al., 2013 [50]<br><br>Iran	Cross-sectional<br><br>To identify the prevalence of differential aspects of fatigue	N=163<br>Sex: Male (61.3%)<br>Age: 61.39±12.61<br>Modality: HD (100%)<br>Time on dialysis (month): 37.6±43.8	Employment: Not reported<br>Income: Poor (19.0%), middle (64.4%), well (16.6%)	Fatigue: Multidimensional Fatigue Inventory (MFI-20)<br>· MFI-20 score: 66.6±15.7, 64.6±16.0 (male and female)<br>· Prevalence of fatigue: 100%	A significant difference was found between reduced activity and motivation dimensions of MFI-20 and employment status (details not provided).

Jhamb et al., 2009 [45]<br><br>USA	Longitudinal - 1 year<br><br>1. To examine the predictors of fatigue at the initiation of dialysis treatment<br>2. To determine the factors related to longitudinal change in vitality<br>3. To examine the relationship of fatigue with HRQOL and survival	N=917<br>Sex: Male (53.1%)<br>Age: 57.9±14.8<br>Modality: HD (100%)<br>Time on dialysis: Not reported	Employment: Unemployed (4.1%)	Fatigue: Medical Outcomes 36-item Short Form (SF-36) - Vitality subscale<br>· SF-36 vitality score: 40.9±22.5	No relevant significant finding.
Jhamb et al., 2011 [46]<br><br>USA	Cross-sectional<br><br>To examine the correlates of fatigue as well as its association with HRQOL, all-cause and cause-specific mortality, and cardiac hospitalizations	N=1,798<br>Sex: Female (56.3%)<br>Age: 57.5±14.0<br>Modality: HD (100%)<br>Time on dialysis: Not reported	Employment: Currently working (9.4%)	Fatigue: Short Form 36-item Short Form (SF-36) - Vitality subscale<br>· SF-36 vitality score: 50.0±21.8	Significant differences in the proportion of currently working patients across levels of fatigue ($p<.001$). However, in multivariate analysis, the association between employment and fatigue was not significant ($p>.02$).
Karakan, Sezer, & Odemir, 2011 [40]<br><br>Turkey	Cross-sectional<br><br>To determine the severity and contributing factors of fatigue	N=154<br>Sex: Male (59.7%)<br>Age: 55 (range not reported)<br>Modality: HD (100%)<br>Time on dialysis (month): 92±65	Employment: Housewife/retired (48.1%), officer/teacher (9.7%), self-employed person (42.2%)<br>Income (duration not specified, US$): <500 (22.1%), 500-2,500 (67.5%), >2,500 (10.4%)	Fatigue: Piper Fatigue Scale (PFS)<br>· PFS score: 1-3 (16%), 4-6 (41%), 7-10 (43%)	Employment status was correlated with the PFS behavior, affective, and total scores (p-values not reported). In multivariate analysis, Unemployment was associated with higher PFS affective (OR: 2.72) and total (OR: 2.25) scores (p-values not reported).
Liu, 2006 [83]<br><br>China (Taiwan)	Cross-sectional<br><br>To test the hypothesized associations between physiological, psychological, and situational variables and level of fatigue	N=119<br>Sex: Female (52.1%)<br>Age: Not reported<br>Modality: HD (100%)<br>Time on dialysis (year): 4.59±4.08	Employment: Yes (42.9%), no (57.1%)	Fatigue: Fatigue Assess Scale (FAS)<br>· FAS energy exhaustion score: 2.45±0.56<br>· FAS decreased mental activity and motivation score: 2.10±0.51<br>· Total FAS score: 2.26±0.49	Patients who were not employed reported higher FAS energy exhaustion, decreased mental activity and motivation, and total scores (all $p<.01$). However, in multivariate analysis, the associations between employment and FAS scores were not significant.
Mollaoglu, 2009 [41]<br><br>Turkey	Cross-sectional<br><br>To examine the level of fatigue and the relationship between the affected factors discussed by people with HD	N=138<br>Sex: Male (56.5%)<br>Age: 48.3±13.4<br>Modality: HD (100%)<br>Time on dialysis (year): 4.63±3.7	Employment: Employed (12.5%), unemployed (87.5%)	Fatigue: Visual Analogue Scale for Fatigue (VAS-F)<br>· VAS-F Fatigue score: 60.7±27.8<br>· VAS-F Energy score: 42.1±11.3	Employed patients reported a lower level of fatigue and a higher level of energy compared with those unemployed ($p<.05$). However, in multivariate analysis, the association between employment and VAS-F score was not significant.

Sesso, Rodrigues-Neto, & Ferraz, 2003 [38]<br><br>Brazil	Longitudinal - 1 year<br><br>To investigate the influence of socioeconomic status on the HRQOL of patients at the beginning of and during the first year of dialysis treatment	N=118 (follow-up: n=119)<br>[Baseline]<br>Age: 49±16<br>Sex: Male (56%)<br>Modality: HD (100%)<br>Time on dialysis (day): 36±15	[Baseline/follow-up]<br>Employment: Employed (37%/36%), unemployed (16%/19%), retired (27%/24%), homemaker (20%/21%)<br>Socioeconomic status (SES): A (8%/7%), B (27%/23%), C (41%/44%), D (19%/20%), E (5%/6%)	[Baseline/follow-up]<br>Fatigue: Medical Outcomes Survey 36-Item Short Form (SF-36) - Vitality pain subscale<br>· SF-36 vitality score: 54±24/50±21<br>Pain: SF-36 - Pain subscale<br>· SF-36 pain score: 65±29/68±30	1. Patients of high SES reported a better SF-36 vitality subscale score (all p<.05) at baseline and follow-up.<br>2. At follow-up, patients of high SES reported a better SF-36 pain subscale score compared with those of low SES (p<.01).<br>3. In multivariate analysis, high SES was a significant predictor of better performance in vitality and pain domains (p-values not reported)
Wang et al., 2016 [30]<br><br>China (Mainland)	Cross-sectional<br><br>To highlight the risk factors of fatigue	N=345<br>Sex: Male (62.6%)<br>Age: 55.6±12.8<br>Modality: HD (100%)<br>Time on dialysis (year): ≥3 (66.4%)	Employment (patients): Employed (15.4%), not employed (84.6%)<br>Employment (children): Employed (64.6%), not employed (20.9%)<br>Economic status: Better (20.9%), normal (43.2%), worse (35.9%)	Fatigue: Functional Assessment of Chronic Illness Therapy-Fatigue (FACIT-F)<br>· FACIT-F score: 39 (31-44)	Patients with worse economic status and those not employed reported a higher FACIT-F score (p≤.006). However, in multivariate analysis, the associations between these factors and FACIT-F score were not significant.
Zuo et al., 2018 [33]<br><br>China (Mainland)	Cross-sectional<br><br>To investigate the characteristics and factors affecting fatigue	N=511<br>Sex: Male (58.5%)<br>Age: 51 (range not reported)<br>Modality: HD (100%)<br>Time on dialysis: Not reported	Employment: Yes (13.1%)<br>Income (monthly, RMB): <900 (12.3%), 901-1,500 (8.4%), 1,501-3,000 (16.0%), 3,001-5,000 (20.5%), >5,001 (42.7%)<br>Medical expense: Own expense (1.2%), medical insurance (75.1%), public expense (1.2%), rural cooperative medical service (22.5%)	Fatigue: Revised Piper Fatigue Scale (RPFS)<br>· RPFS mental fatigue score: 3.63 (0-5.63)<br>· RPFS physical fatigue score: 3.72 (0-5.72)<br>· RPFS score: 3.91 (0-6.09)<br>· Prevalence of fatigue: 61.6%	Unemployed patients and those having income of <RMB900 reported a higher RPFS mental fatigue, physical fatigue, and overall fatigue score (all p<.001). In multivariate analysis, being employed was associated with a lower chance of fatigue (OR: 0.451, p=.012).
Sexual dysfunction					
Anees et al., 2018 [60]	Please refer to 'symptom burden'				
Gatmiri et al., 2018 [54]<br><br>Iran	Cross-sectional<br><br>To investigate the factors affecting sexual function in female HD patients	N=37<br>Age: 49 (25-60)<br>Sex: Female (100%)<br>Modality: HD (100%)<br>Time on dialysis (year): 4.9±4.7, 4.5±6.4 (with and without sexual dysfunction)	Employment: Employed (8.1%), housewife (91.9%)<br>Income (monthly, Rs): <10,000 (16.2%), 10,000-30,000 (81.1%), >30,000 (2.7%)	Sexual dysfunction: Female Sexual Function Index (FSFI)<br>· Prevalence of sexual dysfunction (FSFI ≤ 28): 81.1%	More housewives reported sexual dysfunction (85.3%) compared with those employed (28.6%, p=.05). All patients with income <Rs10,000 reported sexual dysfunction. The proportion is significantly higher than the other groups (p=.05).

Saglimbene et al., 2017 [65]<br><br>5 countries in Europe and South America	Cross-sectional<br><br>To assess the prevalence and correlates of the individual domains of sexual functioning in women treated with HD	N=659<br>Age: 58.8±15.3<br>Sex: Female (100%)<br>Modality: HD (100%)<br>Time on dialysis (month): *41.8 (18.3-76.8)	Employment: Employed (9.1%), unemployed (19.9%), receiving pension (68.7%)	Sexual dysfunction: Female Sexual Function Index (FSFI)<br>· Desire score: *3.6 (3.0-4.2)<br>· Arousal score: *3.9 (3.0-4.8)<br>· Lubrication score: *4.2 (3.6-5.7)<br>· Orgasm score: *4.4 (3.6-5.6)<br>· Satisfaction score: *4.8 (3.6-5.6)<br>· Pain score: *5.0 (4.0-6.0)	Retired and unemployed patients reported lower arousal (OR: -0.49/-0.11) and orgasm scores (OR: -0.63/-0.20, p-values not reported).
Strippoli, 2012 [66]<br><br>27 countries in Europe and South America	Cross-sectional<br><br>To understand the prevalence, severity, and key correlates of sexual dysfunction and to determine whether future research to evaluate health outcomes and screening and intervention strategies is warranted	N=659<br>Age: 58.8±15.3<br>Sex: Female (100%)<br>Modality: HD (100%)<br>Time on dialysis (month): *40 (17.0-77.7)	Employment: Employed (9.3%), unemployed (20.3%), receiving pension (70.3%)	Sexual dysfunction: Female Sexual Function Index (FSFI)<br>· Total FSFI score: *14.8 (2.0-33.1)<br>· Prevalence of sexual dysfunction (FSFI < 26.55): 84.2%	More retired patients reported sexual dysfunction (90.9%) compared with those employed (56.7%) and unemployed (72.5%, p<.001). In multivariate analysis, being unemployed was associated with a higher chance of sexual dysfunction (OR: 2.56, p-value not reported).
Sleep problems					
Einollahi et al., 2015 [51]<br><br>Iran	Cross-sectional<br><br>To determine the prevalence of poor sleep quality and to identify its risk factors in patients on HD	N=6,878<br>Age: 54.4±17.1<br>Sex: Male (57%)<br>Modality: HD (100%)<br>Time on dialysis (month): 13-60 (47.9%)	Employment: Employed (9.8%), unemployed (38.0%), retired (18.2%), housekeeper (30.4%), student (1.0%)	Sleep quality: Kidney Disease Quality of Life-Short Form (KDQOL-SF) - Sleep subscale<br>· KDQOL-SF Sleep score: 55.9±19.9<br>· Prevalence of poor sleep quality (Sleep score > 61.2): 60.5%	More housekeepers reported poor sleep quality (62.8%) and more students reported good sleep quality (64.3%) compared with other groups (<.001). However, in multivariate analysis, the association between employment and sleep quality was not significant.
Rai, Rustagi, & Kohli, 2011 [62]	Please refer to 'depression'				
Trbojević-Stanković et al., 2014 [80]	Please refer to 'depression'				
Zubair & Butt, 2017 [64]<br><br>Pakistan	Cross-sectional<br><br>To investigate the sleep quality and to identify any correlation with the presence of psychiatric morbidity and demographic and social factors	N=140<br>Age: ≤50 (42.9%), >50 (57.1%)<br>Sex: Male (72.1%)<br>Modality: HD (100%)<br>Time on dialysis (year): ≥1 (64.3%)	Employment: Employed (31.4%), unemployed (68.6%)<br>Income: Less than outgoings (63.6%), more than or equal to outgoings (36.4%)	Sleep quality: Pittsburgh Sleep Quality Index (PSQI)<br>· Prevalence of poor sleep quality (PSQI ≥ 5): 68.6%	More patients with income less than outgoings reported poor sleep quality (76.0%) compared with those with income more than or equal to outgoings (24.0%, p<.001). In multivariate analysis, these patients were associated with higher chance of poor sleep quality (OR: 14.029, p<.001).
Pain					

Anees et al., 2018 [60]	Please refer to 'symptom burden'				
Fleishman, Dreiher, & Shvartzman, 2018 [52]<br><br>Israel	Cross-sectional<br><br>To evaluate the prevalence, intensity, and impact of pain, and the subsequent relationships of these factors with demographic and clinical parameters	N=336<br>Age: 63.9±14.8<br>Sex: Male (66.7%)<br>Modality: HD (100%)<br>Time on dialysis (month): ≥24 (52.1%)	Employment: Unemployed (80.4%)<br>Income: Below average (59.8%), average (12.8%), above average (27.4%)	Pain: Leeds Assessment of Neuropathic Symptoms and Signs Pain Scale (S-LANSS)<br>· S-LANSS score: 7.2±2.2<br>· Prevalence of pain: 82%	Patients who were unemployed (p=.001) or had income below average (p=.020) reported a higher level of pain. However, in multivariate analysis, associations between these factors and presence of pain within last 24 hours were not significant.
Sesso, Rodrigues-Neto, & Ferraz, 2003 [38]	Please refer to 'fatigue'				
Constipation					
Zhang et al., 2013 [32]<br><br>China (Mainland)	Cross-sectional<br><br>To evaluate the prevalence of constipation and to determine whether HRQOL is impaired in patients with constipation	N=605<br>Sex: Male (53.9%)<br>Age: 50.0±3.2<br>Modality: HD (79.0%), PD (21.0%)<br>Time on dialysis (month): 53.4±14.9 (HD), 49.6±10.4 (PD)	Employment: Yes (54.7%)<br>Health insurance: Yes (100%)	Constipation: Rome III Criteria<br>· Prevalence of constipation: 59.7%	The proportion of employed patients was lower in the constipated group (33.3%) compared with those non-constipated (88.1%, p<.05). However, this association was not significant in the multivariate analysis.
Itching					
Ersoy & Akyar, 2019 [39]<br><br>Turkey	Cross-sectional<br><br>1. To determine the prevalence of uremic pruritus<br>2. To describe the multidimensional symptom experience<br>3. To determine the associations between pruritus, patient clinical features, and dialysis parameters	N=181<br>Age: 56.9±15.3<br>Sex: Male (52.5%)<br>Modality: HD (100%)<br>Time on dialysis (year): 12.2±6.5	Employment: Employed (4.4%), unemployed (95.6%)	Itching: 5-D Itch Scale<br>· Duration score: 7.2±2.2<br>· Degree score: 3.0±0.8<br>· Direction score: 3.3±0.8<br>· Distribution score: 2.9±0.6<br>· Disability score: 3.8±0.9 (sleep), 2.5±0.8 (social/leisure), 2.4±0.8 (housework), 1.6±0.7 (work/school)<br>· Prevalence of itching: 49.0%	Unemployed patients reported a higher 5-D Itch Scale Duration Dimension score compared with those employed (p=.01).

*Data were reported as median or inter-quartile range.

Abbreviations: HD=Hemodialysis; HRQOL=Health-related quality of life; PD=Peritoneal dialysis
